# Association of inflammatory genes in obstructive sleep apnea and non alcoholic fatty liver disease in Asian Indians residing in north India

**DOI:** 10.1371/journal.pone.0199599

**Published:** 2018-07-12

**Authors:** Surya Prakash Bhatt, Randeep Guleria, Naval K. Vikram, S. V. Nandhan, Yogendra Singh, A. K. Gupta

**Affiliations:** 1 Department of Pulmonary Medicine and Sleep Disorders, All India Institute of Medical Sciences, New Delhi, India; 2 Department of Medicine, All India Institute of Medical Sciences, New Delhi, India; 3 Department of Neuro-Biochemistry, All India Institute of Medical Sciences, New Delhi, India; 4 Department of Radiology, All India Institute of Medical Sciences, New Delhi, India; Auburn University College of Veterinary Medicine, UNITED STATES

## Abstract

**Background:**

Previous studies have indicated that variants of the high sensitive C-reactive protein (*CRP*), Interleukin (*IL*)-6 and leptin receptor (*LEPR*) genes are associated with the presence of obstructive sleep apnea (OSA) but not in non-alcoholic fatty liver disease (NAFLD) in Asian Indians. The study was conducted to investigate the association of *CRP* rs1130864 (1444C/T), *IL-6* rs1800795 (-174G/C) and *LEPR* rs1137101 (Q223R) genes with OSA and NAFLD in Asian Indians residing in North India.

**Methods:**

240 overweight/ obese subjects [body mass index (BMI>23kg/m^2^)], 124 with OSA and with NAFLD (group 1), 47 with OSA without NAFLD (group 2), 44 without OSA and with NAFLD (group 3) and 25 without OSA and without NAFLD (group 4) were recruited in this study. The severity of NAFLD was based on abdomen liver ultrasound and of OSA on overnight polysomnography. Clinical details, anthropometry profile, body composition, biochemical parameters and inflammatory markers were measured. Polymerase chain reaction and restriction fragment length polymorphism of *CRP*, *IL-6* and *LEPR* gene was performed. The associations of these polymorphisms with clinical, anthropometric and biochemical profiles were investigated. The genotypes were confirmed by DNA sequencing analysis.

**Results:**

The C, T and R alleles of *IL-6*, *CRP* and *LEPR* genes was more frequent in OSA and NAFLD subjects and significantly correlated with higher protein levels. The prevalence of variant genotypes C/T of CRP, G/C of IL-6 and Q/R of *LEPR* genes was significantly higher in OSA subjects as compared to non OSA subjects. Further, C/C genotype of IL-6 (G/C), T/T of CRP (C/T) and RR genotype of *LEPR* (Q/R) was associated with significantly higher BMI, fat mass (kg), % body fat, waist circumference, serum triglycerides, total cholesterol, alkaline phosphate, aspartate transaminase and fasting insulin levels in OSA and NAFLD subjects. Using a multivariate analysis, the combined effect of three polymorphisms of *CRP*, *IL-6* and *LEPR* gene variants on OSA and NAFLD risk was evaluated. Odds ratio for OSA and NAFLD with the combination of the three gene polymorphisms increased to 2.84 (95% CI: 1.08–6.54; p = 0.04) even when adjusted for sex, age and BMI.

**Conclusion:**

Polymorphisms of pro-inflammatory cytokine genes were associated with increased risk of OSA and NAFLD in Asian Indians.

## Introduction

Obstructive sleep apnea (OSA) is a complex disease that consists of upper airway obstruction, chronic intermittent hypoxia (CIH) and sleep fragmentation. OSA is associated with insulin resistance, obesity and metabolic syndrome (MS), which is a major risk factor for type 2 diabetes mellitus (T2DM) and cardiovascular disease (CVD). It has been reported that the prevalence of moderate-to-severe sleep-disordered breathing was 23·4% in women and 49·7% in men and 40–70% in obese subjects [1, 2]. Subjects with OSA are predisposed to MS, insulin resistance and impaired lipogenesis [3].

Some authors have suggested that OSA may be another contributor to non-alcoholic fatty liver disease (NAFLD) development. OSA is associated with insulin resistance and hyperlipidemia and both conditions are associated with NAFLD [4]. Previous study indicated that the presence of OSA is also associated with steatosis, necrosis and fibrosis in the liver [5]. OSA and NAFLD have common mechanisms, including insulin resistance and visceral obesity. The relationship between them can be coincidental rather than causal. An animal study showed that the CIH was associated with OSA, steatosis and inflammation in the absence of obesity [6], which suggests that OSA may be a causative factor for NAFLD.

**Fig 1:**
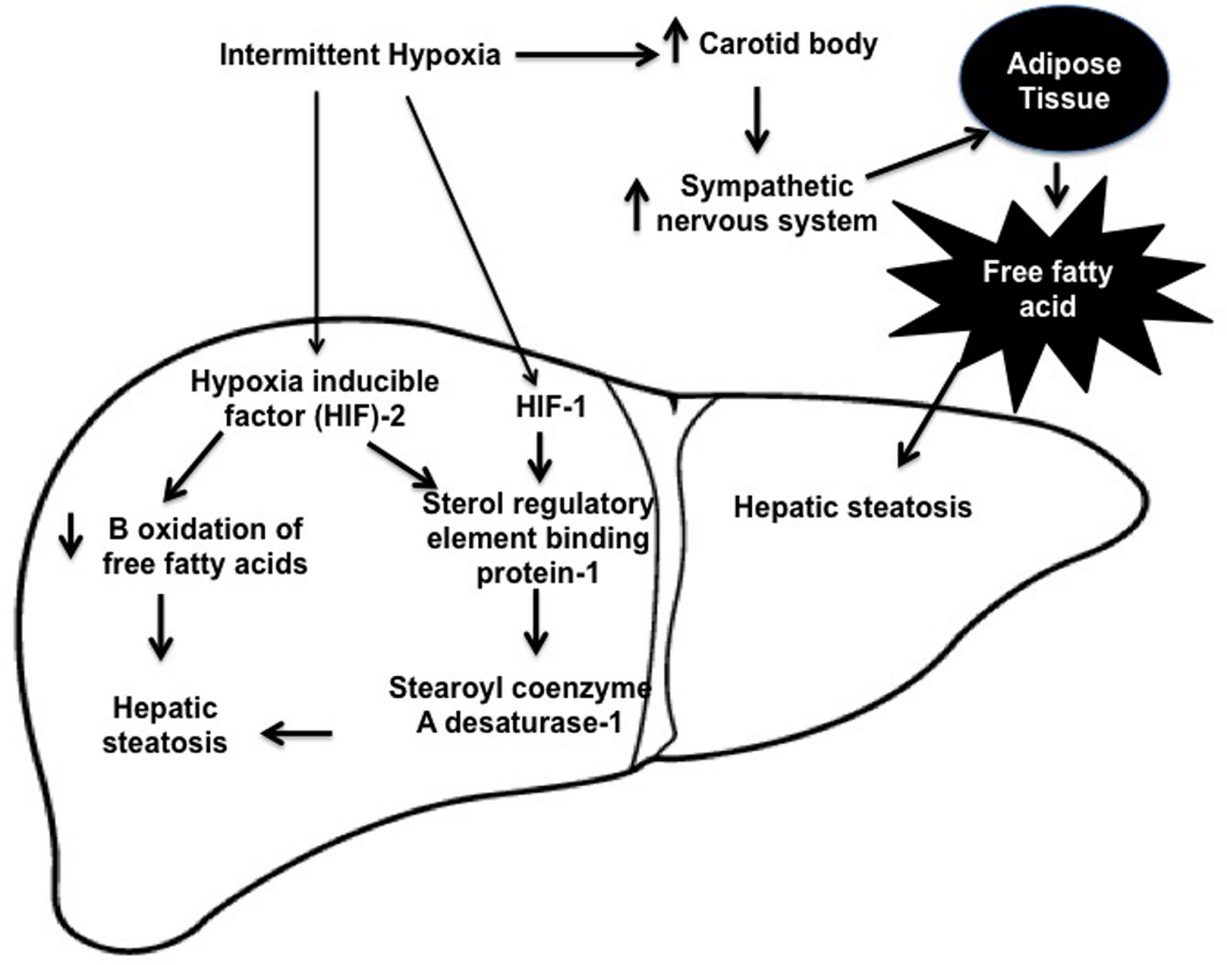
Mechanism of obstructive sleep apnea and non-alcoholic fatty liver disease.

It has been shown that OSA enclosed several physiological mechanisms, which may be predisposed to NAFLD including sleep fragmentation, hypercapnea, and intermittent hypoxia (IH). However, the relationship between NAFLD and IH is presented in Fig 1. In humans OSA, activation of the sympathetic nervous system during IH occurs through hypoxic chemo reflex in the carotid body and ablation of the carotid sinus nerve prevents IH induced hypertension [7]. Jun et al, 2011 [8] have shown that OSA raises circulating free fatty acid (FFA) levels and IH leads to exuberant lipolysis in adipose tissue. The FFA in the liver induces insulin resistance and triglyceride biosynthesis leading to hepatic steatosis [9]. IH induces hepatic steatosis by up-regulating hypoxia inducible factors (HIF) 1 and 2 [10]. The deficiency of HIF-1 alpha completed sterol regulatory element binding protein (SREBP)-1 and stearoyl coenzyme A desaturase (SCD)-1 up-regulation and prevented triglyceride accumulation in the liver during IH [11]. In addition, high levels of serum insulin in obese subjects up-regulate hepatic lipid biosynthesis *de novo* by activating of SREBP-1c [12] and a SREBP-1-regulated enzyme of triglyceride biosynthesis SCD-1 [13].

C reactive protein (CRP), intelukin-6 (IL-6) and leptin are markers of systemic inflammation. We have reported that OSA is a potential driver of elevated CRP levels and this is independent of BMI [14]. IL-6 is an important inflammatory cytokine produce by fibroblasts, endothelial cells, muscle cells, lymphocytes and macrophages. It has been suggested that IL-6 may play an important role in the pathogenesis of atherosclerosis [15]. Further acute-phase proteins can be induced by IL-6 and the higher levels of CRP may increase the production of IL-6 in OSAS patients [16]. Larkin *et al*. [17] showed a significant associations between single nucleotide polymorphisms (SNPs) of *CRP* rs1130864 gene in European American and African American patients with OSA. Studies on *CRP*, IL6 s1800795 gene and leptin receptor (*LEPR*) rs1137101 have been strikingly contradictory in different population.

Asian Indians are predisposed to develop obesity, insulin resistance, MS, NAFLD, OSA, CVD and T2DM. The peculiar dyslipidemic obesity phenotype of Asian Indians with higher truncal and abdominal fat with low lean body mass predisposes this ethnic group to the consequences of a pro-inflammatory and pro-thrombotic state as compared to white Caucasians [18]. The genetic predisposition to OSA and NAFLD in Asian Indians needs more research, and in this context, till date the association of these inflammatory genes in subjects with OSA and NAFLD had not been evaluated In Asian Indians.

We hypothesized that the *CRP* rs1130864 (1444C/T), *IL-6* s1800795 (-174G/C) and *LEPR* rs1137101 (Q223R) polymorphisms which may play an important role in inflammation and this may affect the susceptibility to OSA and NAFLD in non-diabetic Asian Indians. The aim of the study was to assess the effect of the 1444C/T of *CRP*, -174G/C of *IL-6* and 223 Q/R of *LEPR* genes polymorphisms, on the risk of OSA and NAFLD in Asian Indians.

## Methodology

### Subjects

In this study, 240 overweight/ obese subjects [body mass index (BMI>23kg/m^2^)] were evaluated at All India Institute of Medical Sciences, New Delhi, India between May 2012 and January, 2016. 124 with OSA with NAFLD (group 1), 47 with OSA without NAFLD (group 2), 44 without OSA and with NAFLD (group 3) and 25 without OSA and without NAFLD (group 4) were evaluated. The study was approved by the Institutional ethics committee from All India Institute of Medical Sciences, New Delhi, India and written informed consent was obtained from each subject. The inclusion and exclusion criteria’s has been shown in Table 1.

**Table 1:** Inclusion and exclusion criteria.

**Inclusion criteria**	I. BMI > 25 Kg/m^2^ for obese subjects II. Age 18–60 years
**Exclusion criteria**	I. Severe Chronic Obstructive Pulmonary Disease/ advanced lung disease II. Patients having mechanical upper airway obstruction (conformed by ENT consultant) III. Pregnant women IV. Presence of other liver diseases like alcoholic liver disease, viral hepatitis, autoimmune hepatitis, primary biliary cirrhosis, biliary obstruction. V. Drug induced liver damage (e.g. corticosteroids, high-dose estrogens, methotrexate, amiodarone, calcium channel blockers, spironolactone, sulfasalzine, naproxen, or oxacillin, tamoxifen, highly active antiretroviral therapy).

### Clinical and anthropometric measurements

Clinical and anthropometry details were recorded in a sectioned proforma. Blood pressure was measured over the right arm in sitting position. For measurement of weight, height waist circumference (WC), hip circumference (HC), mid thigh circumference (MTC) and skinfold thickness at 6 sites (triceps, biceps, anterior axillary, suprailiac, subscapular and lateral thoracic) were measured according to standard protocols [19].

### Biochemical Investigations

Fasting blood glucose (FBG), lipid profile [20], liver function test [20], fasting insulin [21], Homeostatic model assessment- insulin resistance (HOMA-IR) [21], Hs-CRP [14], IL-6 and leptin levels were estimated (Table 2). Overall, for all the parameters the intra and inter assay percentage coefficient and coefficient of variation were 1.9%, 2.1% and 2.1% and 2.87%, 1.89% and 2.51% for hs-CRP, IL-6 and leptin, respectively.

**Table 2:** Biochemical investigations.

Fasting blood glucose	Randox lab ltd, United Kingdom
Lipid Profile	Total cholesterol, serum triglycerides, high-density lipoprotein cholesterol and low-density lipoprotein cholesterol
Liver function test	Aspartate Aminotransferase, Alanine Aminotransferase, Alkaline Phosphatase
Fasting Insulin and Homeostatic model assessment –insulin resistance	Radioimmunoassay (Immuno tech, France)
Interlukin-6, High sensitive C-reactive protein and leptin levels	Enzyme-linked immunosorbent assay (*Linco* Research Inc., *USA*).

### Ultrasound imaging

All subjects were assessed by a liver ultrasound using 3.5MHz curvilinear probe (Siemens-G 60 S 2004, Germany). For this entire study ultrasound was done by a single radiologist. The definition of fatty liver was based on a comparative assessment of image brightness relative to the kidneys, in line with previously reported diagnostic criteria [22].

### Polysomnography

All patients were called for sleep study at 8.00 pm and were attached to Alice 3 infant and adult computerized polysomnography (PSG) system using the various leads and devices through standard gold cup electrodes [23]. Overnight PSG was measured according to standard protocols [14].

### Genetic analyses

DNA was extracted from the white blood cells using the QIAamp DNA extraction kit (Qiagen, Hilden, Germany) and stored at -20°C for the further experiments. Genotyping was performed by polymerase chain reaction-restriction fragment length polymorphism (PCR-RFLP). Its concentration and quality were then measured in a nanodrop (Thermo Scientific, Waltham, MA, USA). The mean concentration of the samples was 80 to 90 ng/mL. DNA amplification and RFLP of the *CRP* (1444C/T), *IL-6* (-174G/C and *LEPR* (Q223R) gene polymorphisms were performed by previously reported studies [24–26]. The genotypes were confirmed by DNA sequencing analysis. The PCR products were purified by PCR purification kits (QIAGEN, Germany), sequenced in both directions using the Bigdye Termination Kit (Applied Biosystem, Foster City, CA, USA) and analyzed on the ABI Prism 3100 automated sequencer (Applied BioSystems, Foster City, USA) [27]

### Definitions/ Criteria used in this study

Overweight and obesity was defined as BMI 23–24.9 kg/m^2^ and BMI >25 kg/m^2^, respectively according to criteria for Asian Indians [28]. Similarly, WC cut-offs of >90 cm for males and >80 cm for females were considered an indicator of abdominal obesity [20]. FBG<100 mg/dl, serum triglyceride (TG) >150 mg/dl (or on lipid lowering drugs), blood pressure >130/85 mmHg (or on antihypertensive therapy) and high density lipoprotein- cholesterol (HDL-C); males <40 mg/dl, and females <50 mg/dl [20] were defined as abnormal. The modified criteria of National Cholesterol Education Program, Adult Treatment Panel III (NCEP, ATP III) was used to define the metabolic syndrome [29]. Insulin resistance was measured by two surrogate measures: fasting hyperinsulinemia and Homoeostasis Model Assessment of insulin resistance (HOMA-IR). Hyperinsulinemia was defined as values in the highest quartile [30]. The value of HOMA-IR was calculated as = fasting insulin (U/ml) × fasting glucose (mmol/l)/22·5 [31]. Diagnosis of OSA was made on the basis of international classification of sleep disorders (ASDA diagnostic classification steering committee). Breathing event was defined according to the commonly used clinical criteria published by American Academy of Sleep Medicine Task Force [23]. Apnea was defined as cessation of airflow >10 s. Hypopnea was defined as recognizable, transient reduction of breathing >10 s associated with either an oxygen desaturation of >3% or an arousal. AHI was defined as number of obstructive apneas and hypopneas per hour of sleep. Subjects with an AHI < 5/hour were assigned as not having OSA and subjects with an AHI ≥5/hour were diagnosed to have OSA. Polysomnography was conducted in a single sleep laboratory and analysis was done by a single expert. Subjects having OSA were further classified as mild (AHI 5–14.9/h), moderate (AHI 15-30/h) and severe (AHI > 30/h). High serum hs-CRP level was defined as >1 mg/L [14, 32]. The minimum detectable concentration of IL-6 was estimated to be 2 pg/ml. The lowest detectable level of serum leptin was 0.2 ng/ mL.

### Statistical analysis

Data were entered in an Excel spreadsheet (Microsoft Corp, Washington, USA). The distribution of clinical, biochemical, anthropometric and body composition parameters was confirmed for approximate normality. Categorical data were analyzed by Chi-squared test, with Fisher correction when appropriate, and expressed as absolute number (%). Continuous variables were expressed as the mean ± standard deviation to summarize the variables. All continuous values were performed using the Z score method. The influence of the groups (1*vs*2, 1 *vs*3, 1*vs* 4, 2*vs*3 and 2*vs*4) was estimated by the Analysis of covariance (ANCOVA) test with multiple comparisons. Pearson’s correlation coefficient and significance of ‘r’ was used to compare between the inflammatory marker levels and clinical parameters.

In order to determine if observed allele frequency was in conformity with the expected frequency (Hardy Weinberg equilibrium), chi-square analysis was done. Between-group differences in proportions of alleles or genotypes were compared using Chi-square test and a two-tailed Fisher’s exact test. The influence of the genotype on the clinical biochemical, anthropometric and body composition parameters was estimated by ANOVA. Univariate and multivariate analyses were carried out to identify the independent predictors of OSA and NAFLD considering age, sex, BMI and fasting insulin. Bonferroni corrections for multiple comparisons were performed. The odds ratio (OR) and 95% confidence interval were used as a measure of strength for the association between CRP, IL-6 and LEPR genotypic combinations with the disease. A p value <0.05 was considered as significant.

## Results

### Demographic, clinical, anthropometric profiles

Demographic, clinical and anthropometric profiles are presented in Table 3. It was observed that male subjects were higher in all groups but statically not significant. Mean values of blood pressure (systolic and diastolic), BMI, fat mass, fat free mass, %body fat, WC, HC, MTC, neck, subscapular, suprailiac, lateral thoracic and thigh were significantly higher in OSA with NAFLD group as compared to the other groups.

**Table 3:** Clinical, body composition and anthropometry investigations.

Variables		Group 1 (n = 124)	Group 2 (n = 47)	Group 3 (n = 44)	Group 4 (n = 25)	P value						
						Total	1&2	1&3	1&4	2&3	2&4	3 & 4
Age (yrs)		44.8±9.1	44.2±9.1	39.5±10.5	41±8.5	0.08	0.3	0.02	0.06	0.05	0.07	0.05
Sex, n (%)	Male	64 (51.6)	25 (53.2)	28 (63.6)	13 (52)	0.06	0.3	0.2	0.6	0.6	0.08	0.4
	Female	60 (48.4)	22 (46.8)	16 (36.4)	12 (48)	0.06	0.4	0.1	0.5	0.3	0.09	0.5
SYBP (mmHg)		131.4±11.5	130.2±15.6	126±20.6	120±16.9	0.001	0.2	0.08	0.01	0.07	0.04	0.04
DY BP (mmHg)		84.4±14.4	83.5±13.6	82.2±15.4	80.1±13.6	0.003	0.1	0.09	0.05	0.2	0.05	0.3
Pulse Rate		79.70±7.8	79.71±5.9	76.6±5.1	76.83±4.6	0.11	0.3	0.6	0.8	0.8	0.7	0.9
BMI (Kg/m2)		33.3±7.9	32.5±6.9	31.0±8.3	28.5±8.6	0.003	0.09	0.06	0.003	0.1	0.05	0.05
Fat mass (kg)		40.45±17.4	35.6±14.2	31.1±14	30.5±9.5	0.02	0.04	0.05	0.01	0.1	0.2	0.3
Fat free mass (kg)		54.1±12.1	52.4±11.7	48.03±12.1	45.7±9.7	0.002	0.05	0.02	0.01	0.6	0.04	0.5
Total Body Water (kg)		40.6±8.6	38.2±9.6	35.2±8.7	33.2±8.3	0.5	0.4	0.6	0.06	0.4	0.09	0.7
Body fat (%)		40.2±13.6	38.2±11.6	37.1±12.8	34.6±11.6	0.002	0.1	0.05	0.002	0.9	0.04	0.04
WC (cm)		106.9±13.3	104.2±14.6	102±13.5	100±15.6	0.001	0.7	0.03	0.001	0.8	0.03	0.08
HC (cm)		109.5±13.2	106.5±23.5	103±16.9	101.7±15.9	0.003	0.04	0.02	0.003	0.07	0.01	0.02
MTC (cm)		55.8±7.4	54.1±8.6	53.2±8.9	52±7.9	0.005	0.1	0.09	0.005	0.3	0.9	0.8
Mid Arm		32.16±7.6	30±6.5	29.7±6.4	24.6±5.6	0.6	0.9	0.8	0.05	0.9	0.07	0.4
Neck		38.74±5.6	38.3±3.6	36.2±4.04	32.1±3.1	0.0004	0.8	0.2	0.002	0.8	0.07	0.08
Biceps		16.8±7.07	15.4±6.7	17.36±5.4	15.2±5.5	0.9	0.9	0.6	0.5	0.8	0.2	0.9
Triceps		25.0±10.5	24.3±9.3	24.3±7.7	22.2±9.3	0.44	0.6	0.5	0.9	0.8	0.6	0.4
Subscapular (mm)		30±8.1	29.2±9.8	27±5.6	26±6.5	0.2	0.8	0.6	0.8	0.4	0.1	0.6
Ant axillary		17±6.0	14.6±5.3	13.27±5.2	13.7±5.1	0.5	0.21	0.3	0.8	0.9	0.5	0.6
Suprailiac (mm)		31.8±9.8	29.2±10.5	28.1±9.5	27±8.9	0.02	0.05	0.01	0.02	0.9	0.1	0.6
Lateral thoracic (mm)		33.7±11.1	31.9±12.5	30.1±11.9	28.9±9.8	0.02	0.07	0.07	0.02	0.6	0.07	0.09
Thigh		30.1±11.3	26±9.9	25.4±6.6	24.7±8.1	0.05	0.07	0.05	0.02	0.6	0.8	0.7

Results are shown as mean± SD. P value is <0.05 is statistically significant. SYBP, systolic blood pressure; DYBP, diastolic blood pressure; BMI, body mass index; WC, waist circumference; HC hip circumference; MTC, mid thigh circumference.

### Biochemical profiles

The biochemical investigations of the four groups are given in Table 4. It was observed that the mean values of FBS, TG, total cholesterol (TC), HDL, low density lipoprotein (LDL), aspartate aminotransferase (AST), alanine aminotransferase (ALT), alkaline phosphatase (ALP), fasting Insulin and HOMA-IR were significantly increased in OSA with NAFLD group as compared to controls. Mean values of hs-CRP, IL-6 and leptin levels were significantly increased in OSA with NAFLD group as compared to controls (p<0.05).

**Table 4:** Biochemical profiles of the subjects.

Variables	Group 1 (n = 124)	Group 2 (n = 47)	Group 3 (n = 44)	Group 4 (n = 25)	P value						
					Total	1&2	1&3	1&4	2&3	2&4	3 & 4
FBS	103±25.2	104.1±38.4	98.14±21.2	90.3±24.4	0.004	0.4	0.001	0.001	0.003	0.002	0.07
TG (mg/dl)	189±40.6	177±46.9	158.1±55.2	151±58.9	0.01	0.005	0.0002	0.001	0.4	0.04	0.02
T.C (mg/dl)	185±38.3	180±44.6	178±43.6	171±39.8	0.02	0.004	0.006	0.0001	0.2	0.04	0.003
HDL (mg/dl)	42.4±8.3	43.8±11.7	44.6±9.1	52.3±10.2	0.005	0.5	0.6	0.02	0.8	0.06	0.05
LDL	112.6±40.2	109±36.5	109±35.6	98±30.8	0.002	0.6	0.5	0.04	0.9	0.04	0.04
VLDL	33.5±11.2	32±12.3	31±11.3	30.0±9.6	0.4	0.6	0.5	0.7	0.9	0.2	0.3
AST (IU/L)	44.5±15.9	41.4±22.1	39.6±19.6	31.6±15.9	0.01	0.7	0.03	0.001	0.05	0.02	0.001
ALT (IU/L)	60.9±10.3	54.2±12.9	52.3±11.9	50.9±10.9	0.03	0.03	0.04	0.01	0.09	0.05	0.3
ALP (IU/L)	240.6±74.3	242±76.5	235±72.9	235±69.8	0.05	0.1	0.05	0.05	0.06	0.08	0.8
Insulin (μU/ml)	12±4.3	11.1±4.8	9.3±3.6	9.3±3.8	0.001	0.05	0.03	0.001	0.02	0.004	0.09
HOMA-IR	2.9±0.92	2.5±0.98	1.9±0.86	1.6±0.76	0.001	0.05	0.03	0.001	0.1	0.002	0.5
IL-6 (pg/ml)	20.1±5.6	17.5±6.5	16.5±4.6	15.4±3.5	0.05	0.6	0.05	0.03	0.3	0.05	0.2
Hs -CRP (mg/L)	4.2±2.2	3.6±1.5	3.1±1.3	1.4±0.7	0.0001	0.05	0.006	0.0001	0.6	0.003	0.01

Results are shown as mean± SD. P value is <0.05 is statistically significant; FBG, fasting blood glucose; TG, triglyceride; TC, total cholesterol; HDL, high density lipoprotein; LDL, low density lipoprotein, VLDL, very low density lipoprotein; ALP, Alkaline phosphate; ALT, alanine transaminase; AST, aspartate transaminase; HOMA-IR, homoeostasis modal assessment for insulin resistance, IL-6, Interleukin 6; hs-CRP, high-sensitivity C-reactive protein

A correlation analysis of CRP, IL-6 and leptin with other variables was investigated. In OSA and NAFLD group, hs-CRP levels significantly correlating with BMI (r = 0.6059, p = 0.002), fat mass (r = 0.5949, p = 0.05), % body fat (r = 0.5831, p = 0.04) and IL-6 (r = 0.6071, p = 0.005) levels. IL-6 levels significantly correlating with % body fat (r = 0.6138, p = 0.004), fasting insulin (r = 0.6135, p = 0.005), hs-CRP (r = 0.6071, p = 0.005) levels in OSA and NAFLD group. Leptin levels significantly correlating with BMI (r = 0.6159, p = 0.003), % body fat (r = 0.6089, p = 0.002), fasting insulin (r = 0.5968, p = 0.005), IL-6 (r = 0.5938, p = 0.005) and CRP (r = 0.6038, p = 0.001) in OSA and NAFLD group.

### Genotype distribution

**Fig 2:**
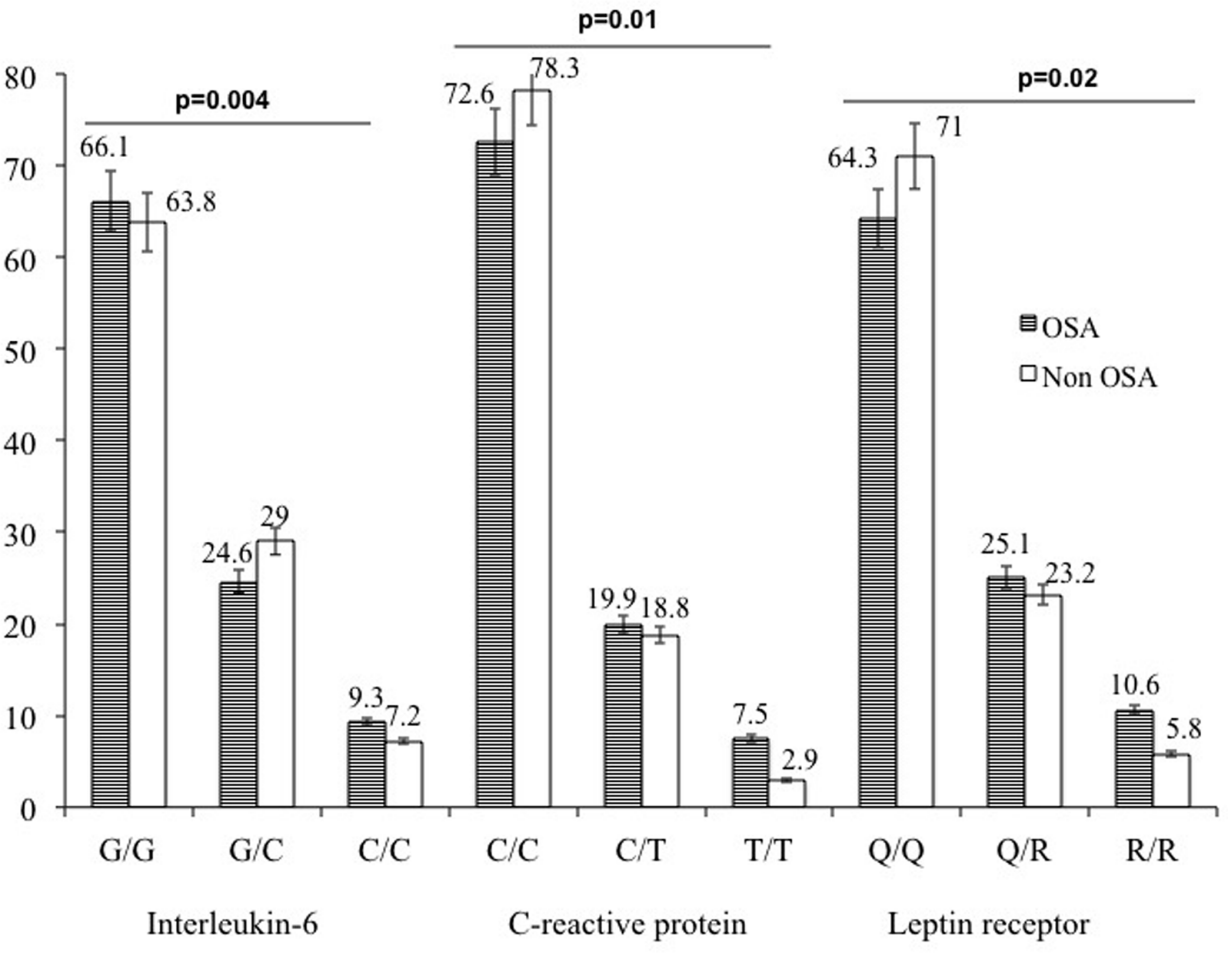
Genotypic frequencies of Interleukin 6, high-sensitivity C-reactive protein and leptin receptor genes with OSA (group-1), without OSA (group-2).

Genotypic data has been categorized in two group; OSA and non OSA. The reproducibility of the genotype data was checked and compared by replication the genotype in 120 subjects by sequencing the different genotype. The frequency of the *CRP*, *IL-6* and *LEPR* gens was presented in Fig 2.

*IL-6* (-174 G/C): Association of IL-6 gene polymorphism with clinical, body composition, anthropometry and biochemical parameters was presented in Table 5. The Il-6 Hardy Weinberg equilibrium p-value and chi values in OSA and non OSA subjects were p = 0.006, chi value = 13.54 and p = 0.14, chi value = 2.149 respectively. In OSA subjects, BMI, fat mass, % body fat, WC, thigh, lateral thoracic, FBS, TG, TC, ALT, AST, ALK, fasting insulin, hs-CRP, and serum IL-6 levels were significantly increased in G/C genotype as compared to G/G genotype. In group 2, BMI, fat mass, % body fat, FBS, TG, TC, LDL, ALT, AST, ALK and serum IL-6 levels were significantly increased in C/C genotype as compared to G/G and G/C genotypes. In Non OSA subject, BMI, FFM, serum TG, TC, LDL, AST and serum IL-6 levels were significantly increased in C/C genotype as compared to G/G and G/C genotypes.

**Table 5:** Association of IL-6 gene polymorphism with clinical, body composition, anthropometry and biochemical parameters.

Variables	OSA (n = 171)			Non OSA (n = 69)		
	G/G	G/C	C/C	G/G	G/C	C/C
Numbers (%)	113 (66.1)	42(24.6)	16(9.3)	44(63.8)	20 (29)	5(7.2)
Age (yrs)	45.0±9.1	46.3±9.8	47.3±10.0	41.0±10.5	39.1±15.1	40.9±15.4
SYSBP (mmHg)	132.5±12.3	133.1±11.1	134.5±14.1	129.7±9.3	131.1±18.6	132.7±18.6
DSBP (mmHg)	85.9±8.1	84.3±12.3	85.9±18.3	85.4±10.1	83±5.1	84.6±5.4
BMI (kg/m^2^)	36.5±13.0	37.9±12.3	41.8±14.2*	30.1±6.7	33±7.3	34.1±7.7*
Fat mass (kg)	37.1±17.5	40.3±18.5	46.3±94.5*	31.0±14.0	32.1±16.1	33.3±17.5
FFM (kg)	54.4±12.4	53.1±11.9	53.4±11.7	45.3±11.7	51.1±11.1	52.0±11.9*
Body fat (%)	35.9±12.0	39.5±9.9	40.6±8.9*	36.9±14.1	37±12.1	39.9±12.8
WC (cm)	104.9±15.5	106.4±12.1	108.4±13.9*	100.8±10.4	106±12.0	107±14.0
HC (cm)	105.2±16.8	108±14.2	110.9±16.6	102.8±9.4	106±15.1	107.0±15.3
MTC (cm)	55.8±13.4	57.2±12.1	57.9±13.8	51±11.7	52±11.1	54.1±11.8
MAC (cm)	28.5±6.1	29.8±7.9	30.7±6.7	28.4±5.1	24.2±5.0	28.2±5.0
NC (cm)	37.5±7.7	38.0±4.1	39.3±4.1	36.9±3.9	32.6±4.0	35.6±4.0
Biceps (mm)	15.8±6.5	16.7±7.1	17.3±7.3	13.2±6.0	14.8±5.7	15.8±5.7
Triceps (mm)	23.8±9.7	24.5±9	25.6±10.1	20.1±7.6	22.2±10.8	25.2±10.8
Sub Scapular (mm)	28.6±8.9	28.9±8.2	30.6±8.2	25.3±8.3	24.5±9.2	29.5±10.2
Anti axillary (mm)	17.6±5.9	17.9±5.9	16.3±5.9	11.6±4.5	14.1±4.5	15.5±5.5
Suprailiac (mm)	30.5±9.4	31.4±8.1	32.5±8.4	27.8±7.9	31±7.4	32.0±8.4
Lateral Thoracic	37.3±28.5	40.4±74.1	43.5±74.1*	30.5±9.0	32.1±10.1	35.3±11.1
Thigh (mm)	26.2±8.8	30.9±8	33.8±8.7*	23.3±8.6	24.5±6.1	26.7±7.1
FBG (mg/dl)	100.5±15.9	109±41.7	110.8±45.2*	97.2±25.3	97.2±22.5	99.6±12.5
TG (mg/dl)	155.6±72.6	175.3±80	184.3±91.0*	157.2±39.9	166±98.2	177.2±99.2*
TC (mg/dl)	173.0±44.01	188.6±34.5	194.5±49.6*	171.3±28.3	193.9±45.5	192.9±45.5*
HDL-C (mg/dl)	41.2±7.9	40.1±8.3	43.0±8.3	45.7±10.6	41±6.0	42.8±6.0
LDL-C (mg/dl)	115.2±45.5	116.8±39.5	117.4±39.5	98.2±36.4	105.2±37.3	107.8±37.3*
VLDL (mg/dl)	29.5±13.7	29.8±12.1	31.1±12.1	30.8±9.	31.2±10.0	32.4±10.0
ALT (mg/dl)	39.8±30.5	40.1±30.5	43.5±30.5*	40.1±22.6	41.9±16.3	40.6±16.3
AST (IU/L)	45.6±30.5	46.8±5.8	50.2±5.8*	49.6±36.3	50.2±44.2	52.6±44.2*
ALK (IU/L)	196.6±72.1	205±75.7	207.8±80.6*	180.8±89.8	185±91.7	191.1±91.7
Insulin (μU/ml)	10.3±7.0	11.8±5.8	12.6±7.4*	8.8±3.9	10.0±4.5	10.0±4.5
IL-6(pg/ml)	14.2±9.2	20.9±7.1	23.2±7.1*	14.4±7.9	16.7±9.0	18.9±9.0*
Hs-CRP (mg/L)	3.1±1.9	3.5±2.0	4.1±2.0*	1.35±0.9	2.11±1.0	2.36±1.0

Results are shown as mean± SD.

*P value is <0.05 is statistically significant. SYBP, systolic blood pressure; DYBP, diastolic blood pressure; BMI, body mass index; FFM, fat free mass; WC, waist circumference; HC hip circumference; MTC, mid thigh circumference; MAC, mid arm circumference; NC, neck circumference; FBG, fasting blood glucose; TG, triglyceride; TC, total cholesterol; HDL, high density lipoprotein; VLDL, very low density lipoprotein; ALP, Alkaline phosphate; ALT, alanine transaminase; AST, aspartate transaminase; IL-6, Interleukin-6.

*CRP* (1444C/T): The *CRP* Hardy Weinberg equilibrium p-value and chi values in OSA and non OSA subjects were p = 0.0019, chi value = 9.564 and p = 0.19, chi value = 1.65 respectively. Association of CRP gene with metabolic and body composition parameters was shown in Table 6. OSA subjects, BMI, fat mass, fat free mass, % body fat, WC, HC, MTC, subscapular, thigh, TG, TC, LDL, very low density lipoprotein- cholesterol (VLDL-C), ALT, AST, ALK, fasting insulin, hs-CRP and IL-6 levels were significantly increased in T/T genotype as compared to other genotypes. In non OSA subject, fat free mass, % body fat, MTC, TG, TC, LDL, ALT and ALK levels were significantly increased in C/T genotype as compared to C/C genotype.

**Table 6:** Association of CRP gene polymorphism with clinical, body composition, anthropometry and biochemical parameters.

Variables	OSA (n = 171)			Non OSA (n = 69)		
	C/C	C/T	T/T	C/C	C/T	T/T
Numbers (%)	124 (72.6)	34 (19.9)	13 (7.5)	54 (78.3)	13(18.8)	2 (2.9)
Age (yrs)	44.7 ±9.2	44.5±8.5	46.2±9.5	40.7±13.0	41.2±9.9	42.2±10.9
SBP (mm	133.1±11.8	133.4±11.8	133.6±15.8	131.5±16.6	130.4±7.2	131.5±7.5
DBP (mmHg)	85.6±7.4	85.9±17.9	86.9±20.9	85.0±8.7	84.2±7.3	85.1±7.6
BMI (kg/m^2^)	35.5±17.3	37.8± 23.1	39.1± 37.1*	31.2±6.9	33.8±8.3	34.6±9.3
Fat mass (kg)	35.5±17.0	40±16.9	53.0±16.1*	35.1±16.4	36.9±12.9	37.9±14.8
FFM (kg)	56.8±11.7	54±13.1	50.6±13.2*	54.1±9.2	50.3±9.0	46.2±11.9*
Body fat (%)	37.3±11.7	41.2±12.1	41.2±12.3*	38.6±15.0	40.1±11	41.2±13.0*
WC (cm)	105.3±13.7	108.3±12.43	109.3±12.4*	103.1±12.2	103.9±13.1	104.5±13.6
HC (cm)	106±17.4	109.0±16.4	110.0±16.6*	103.4±10.4	105±13.8	106.1±19.4
MTC (cm)	54.2±6.3	55.7±14.5	59.7±14.7*	47.5±9.6	51.0±11	53.8±12.00*
MAC (cm)	31.4±5.6	31.4±19.2	32.4±19.6	28.3±4.8	28.9±6.2	29±6.5
NC (cm)	38.8±6.3	37.9±3.3	38.5±3.6	36.1±3.99	38.7±6.0	36.5±4.6
Biceps (mm)	16.2±6.5	18±7.4	19±7.6	16.7±20.3	18.0±9.8	20.8±10.4
Triceps (mm)	24.5±9.4	26.2±10.4	26.2±11.4	20.6±8.6	24.7±10.1	25.6±9.9
Sub Scapular	29.5±8.4	34.2±3.7	35.2±4.8*	26.3±9.3	28.9±8.3	30.8±8.5
Anti axillary (mm)	16.1±5.4	17.1±4.7	18.5±6.8	12.6±5.0	14.9±5.1	16.5±5.5
Suprailiac (mm)	31.5±8.8	31.1±8.2	32.4±8.8	29.5±8.7	29.8±5.6	29.4±6.5
Lateral Thoracic	31.3±9.2	32.5±17.1	36.5±27.5*	32.3±10.2	32.9±10.2	33.1±10.2
Thigh (mm)	25.7±9.1	33±3.6*	33±3.6*	24.6±8.7	24.9±4.1	25.3±4.2
FBG (mg/dl)	103.5±24.1	103.4±42.1	106.4±42.3	95.9±19.2	96.9±22.1	97.3±23.2
TG (mg/dl)	156.1±67.4	207.4±95.1	226.4±96.6*	162±49.2	169.0±78.1	171.4±88.7*
TC (mg/dl)	169.7±48.1	176.1±42.2	187.9±42.9*	170.1±27.4	175.9±22.6	182.6±23.7*
HDL-C (mg/dl)	42.7±7.9	43.5±12.1	45.5±12.7	41.6±9.0	42.4±9.1	43.6±9.4
LDL-C (mg/dl)	104.5±42.0	111.1±42.1	116.1±42.8*	103.8±12.4	109.3±38	110.3±39*
VLDL (mg/dl)	29.9±13.1	38.9±13.1	42.9±13.4*	31.9±17.9	31.4±10.3	31.4±10.1
ALT (IU/L)	38.08±20.5	43.4±46.1	48.4±46.5*	37.5±13.1	39.1±17.4	40.7±18.4*
AST (IU/L)	46.6±30.6	51.3±18.1	53.3±19.3*	40.6±14.4	42.3±32.1	44.3±33.5
ALK (IU/L)	191.2±108.2	207.1±75.1	216.1±75.8*	156.2±96.8	165.8±80.1	176.1±86.1*
Insulin (μU/ml)	10.2±2.8	12.1±18.1	12.6±18.8*	9.2±4.0	10.3±5.1	11.4±5.8
L-6(pg/ml)	16.0±9.3	20.8±13.1	21.8±14.9*	16.0±15.2	17.5±8.1	18.0±8.4
Hs-CRP (mg/L)	3.5±1.02	3.9±2.2	4.5±2.10*	1.56±0.09	1.90±1.0	2.56±1.0

Results are shown as mean± SD.

*P value is <0.05 is statistically significant. SYBP, systolic blood pressure; DYBP, diastolic blood pressure, WC, waist circumference; BMI, body mass index; FFM, fat free mass; HC hip circumference; MTC, mid thigh circumference; MAC, mid arm circumference; NC, neck circumference; FBG, fasting blood glucose; TG, triglyceride; TC, total cholesterol; HDL, high density lipoprotein; VLDL, very low density lipoprotein; ALP, Alkaline phosphate; ALT, alanine transaminase; AST, aspartate transaminase; IL-6, Interleukin-6.

*LEPR* (Q223R*)*: Association of *LEPR* polymorphism with clinical and other parameters was shown in Table 7. The *LEPR* Hardy Weinberg equilibrium p-value and chi values in OSA and non OSA subjects were p = 0.003, chi value = 8.67 and p = 0.05 chi value = 3.72 respectively. In OSA subjects, fat mass, % body fat, WC, HC, mid thigh, subscapular, lateral thoracic, TG, TC, ALT, AST, ALK, leptin and IL-6 levels were significantly increased in Q/R genotype as compared to Q/Q genotype. In group 2, systolic blood pressure, fat mass, WC, HC, subscapular, FBS, TG, TC, LDL-C, AST and ALK levels were significantly increased in R/R genotype as compared to other genotypes. In Non OSA subjects, TG, TC, LDL-C, ALT, AST, ALK and IL-6 were significantly increased in R/R genotype as compared to Q/Q and Q/R genotypes.

**Table 7:** Association of LPR gene polymorphism with clinical, body composition, anthropometry and biochemical parameters.

Variables	OSA (n = 171)			Non OSA (n = 69)		
	Q/Q	Q/R	R/R	Q/Q	Q/R	R/R
Numbers (%)	110 (64.3)	43 (25.1)	18(10.6)	49 (71)	16 (23.2)	4 (5.8)
Age (yrs)	45.6±9.0	452±10.6	46.2±10.1	41.1±13.0	41.7±11	40.7±11.8
SYBP (mmHg)	133.6±12.9	133.2±13.1	132.6±13.1	132.0±17.5	131.1±8.3	130.1±8.6
DYSBP (mmHg)	86.3±8.3	85.9±11.3	85.4±17.5	85±9.1	84±6.4	85.3±6.9
BMI (kg/m^2^)	38.0±14.5	39.4±12.9	39.1±11.0	31.7±6.9	32±8.1	31.9±8.6
Fat mass (kg)	38.1±17.1	40.7±9.3	44.3±9.6*	36.2±16.6	33.1±15.1	34.1±15.2
FFM (kg)	54.5±13.0	50.2±10.1	53.2±10.7	46.8±12.7	48.5±10.2	50.9±10.3
Body fat (%)	35.9±12.0	36.5±10.1	38.5±11.7*	39.0±11.5	40.1±14.1	41.2±14.2
Circumferences (cm)						
WC	104.7±11.4	106±14	108.6±16.0*	101.7±13.6	102±11.1	104±11.9
HC	105.9±11.6	107±14.2	110.7±19.2*	104.6±11.1	103.1±16.2	104.3±15.2
MTC	55.6±12.7	56.7±12.1	58.1±14.2*	51.1±10.6	52.1±12.1	53.4±12.2
MAC	28.3±5.0	30.7±7.1	30.9±7.2	28.2±5.0	29.7±5.2	28.9±5.3
NC	37.5±6.8	38.1±4.1	39.4±4.7	36.0±4.2	35.8±3.1	36.5±3.6
Skinfolds (mm)						
Biceps	17.7±7.2	15.9±6.5	15.4±6.5	14±6.5	17.8±21.0	18.6±22.0
Triceps	22.9±9.10	25.2±9.8	26.2±10.3	22.2±9.4	21.5±9.1	22.2±9.2
Sub Scapular	28.1±8.9	29.8±7.1	31.0±8.1*	26.1±9.6	25.9±9.1	27.4±9.2
Anti axillary	16.9±5.7	17.3±6.1	16.5±6.3	13.3±5.4	12.9±4.2	13.0±4.9
Suprailiac	30.2±9.2	31.2±8.2	32.8±8.4	27.8±7.6	29.1±8.3	30.1±8.6
Lateral Thoracic	31.3±10.1	40.5±6.1	44.5±61.8*	30.7±9.6	31.5±10.2	33.1±10.4
Thigh (	26.4±9.6	24.5±10.1	25.0±10.4	25.3±8.9	24.7±6.3	23.5±6.0
FBG (mg/dl)	103.8±24.6	102.4±26.1	103.1±26.5	98.6±24.6	97±11.2	96.9±10.6
TG (mg/dl)	150.9±70.4	167±70.4	172.4±77.1*	172.4±94.8	168±40.8	163.5±46.5*
TC (mg/dl)	179.5±53.4	180±36.1	182.0±37.2*	165.6±24.5	183.0±41.2	193.0±44.0*
HDL-C (mg/dl)	42.6±8.2	41.6±8.1	41.9±8.2	43±4.7	44±10.1	45.3±10.5
LDL-C (mg/dl)	113.9±47.4	111±34	110.4±34.2	95.3±21.5	101±38.4	115.1±39.9*
VLDL (mg/dl)	29.4±13.9	30.3±11.1	31.1±11.8	29.4±9.4	34.7±18.2	32.8±19.1
ALT (IU/L)	39.3±25.3	40.10±32.1	42.0±33.1*	36.3±16.9	39.0±20.2	42.0±21.6*
AST (IU/L)	45.5±31.6	49.4±32.0	50.0±32.1*	47.1±41.4	49.0±40.1	53.4±41.4
ALK (IU/L)	177.8±89.4	186.2±89.9	195.4±91.5*	182.7±58.0	201.5±78.2	212.5±82.9
Insulin (μU/ml)	10.8±6.9	11.5±7.3	12.5±7.5	8.5±4.2	9.1±4.1	9.6±4.2
Leptin (ng/ml)	17.9±9.3	19.3±11.1	21.3±10.3*	19.8±11.7	19.8±8.1	20.3±8.5
Adiponectin (μg/ml)	14.2±6.3	13.2±5.3	12.4±5.5	13.5±5.8	13.2±3.1	13.5±3.5
IL-6 (pg/ml)	15.5±9.3	21.0±8.2*	23.0±7.6*	11.2±6.3	19.3±8.1	19.4±8.6*

Results are shown as mean± SD.

*P value is <0.05 is statistically significant. SYBP, systolic blood pressure; DYBP, diastolic blood pressure, WC, waist circumference; BMI, body mass index; FFM, fat free mass; HC hip circumference; MTC, mid thigh circumference; MAC, mid arm circumference; NC, neck circumference; FBG, fasting blood glucose; TG, triglyceride; TC, total cholesterol; HDL, high density lipoprotein; VLDL, very low density lipoprotein; ALP, Alkaline phosphate; ALT, alanine transaminase; AST, aspartate transaminase; IL-6, Interleukin-6.

### Interaction between *CRP*, *IL-6* and *LEPR* genotypes

The genotype interaction between *CRP*, *IL-6* and *LEPR* was analyzed. It was observed that the prevalence of variant genotypes *T/T* of *CRP*, *G/C* of *IL-6 and Q/R of LEPR* genes was significantly higher in OSA and NAFLD group. We evaluated the combined effect of these three polymorphisms of *CRP*, *IL-6* and *LEPR* variants on OSA and NAFLD risk using multivariate analysis. ORs for OSA and NAFLD with the combination of the three gene polymorphisms increased to 2.84 (95% CI: 1.08–6.54; p = 0.04) even when adjusted for sex, age BMI and fasting insulin.

## Discussion

In this study, which has not been done before, we observed that *CRP*, *LEPR* and *IL-6* polymorphisms was at a significantly high risk of developing OSA and NAFLD. These finding in Asian Indians may imply that the inflammatory gene (*CRP*, *IL-6* and *LEPR*) variants are closely linked to OSA and NAFLD disease mutations. The most of the published literatures on genetics of OSA has mainly focused on other disease but not on OSA and NAFLD. These observations in Asian Indian population may imply that the IL-6/CRP pathway is causally associated with OSA and NAFLD or alternatively the IL-6/CRP gene variants are tightly linked to the true disease mutations. Previous report has been indicated that during nocturnal hypoxia, adipocytes and monocytes secrete IL-6 through the nuclear factor-*κ*B pathway. IL-6 describe an important encouragement of CRP production in the liver [33] and has an important role in inflammatory processes in OSA. The combined data of genetic association of OSA and NAFLD is not available in Asian Indians.

Asian Indian population is known to have a lower muscle mass and higher body fat, abdominal obesity, insulin resistance and metabolic syndrome than the white population [34]. It has been suggested that all factors associated with OSA and NAFLD such as generalized obesity, abdominal obesity, T2DM, hyper-triglyceridaemia, MS and insulin resistance are more prevalent in Asian Indians [35]. Previous study on serum leptin, IL-6 and hs-CRP levels have been conducted in acute myocardial infarction (AMI) patients from south India (Chennai) [36]. That study was carried out on 195 subjects and reported a strong correlation between serum levels of leptin, IL-6 and AMI. This suggests an involvement of leptin in the up-regulation of inflammatory cytokines during AMI. Our group has reported that obese subjects with OSA had significantly higher CRP levels then non OSA obese subjects. These levels were proportional to the increase in severity of OSA [14]. Furthermore, the present study is also shows that a strong positive correlation between serum levels of hs CRP, leptin and IL-6 with OSA and NAFLD. Serum leptin was found to be correlated with hs-CRP values in the OSA and NAFLD group. Such association has not been identified in Asian population previously. It appears therefore that leptin concentration is translated into changes in IL-6 concentration possible at the level of IL-6 gene expression. Although there is a strong relationship between OSA and proinflammatory cytokines such as leptin, CRP and IL-6, the underlying mechanisms of this relationship remain unclear. Hypoxia, manifested as the recurrence of desaturation is probably the main contributor to inflammation in OSA [37]. Previous study has been reported positive association between leptin, hs-CRP and IL-6 [38]. Haddy *et al*. reported that there was a strong correlation between IL-6 and CRP [39]. Therefore, it may be speculated that IL-6 is not only a potent hepatic stimulus for CRP but also associated with increased tissue levels in atherosclerotic plaque. Karaduman *et al* [40] showed that the levels of leptin, hs-CRP and sIL-6R were significantly higher in patients with diabetes mellitus than without diabetes mellitus. Consequently, our findings confirm that leptin, along with other inflammatory markers such as hs-CRP and sIL-6R, may contribute to the progression of OSA and NAFLD.

The polymorphisms of *CRP*, *IL-6* and *LEPR* genes were significantly associated with OSA and NAFLD. A notable finding of this study is a significant interaction (epistasis) between the three gene variants. Indeed, subjects carrying the variants of the three polymorphisms had an adjusted OR of 2.84 (95% CI: 1.08–6.54; p = 0.04) for the risk of OSA and NAFLD. Further, we observed a strong association of variant genotypes with body fat and metabolic parameters. Previous candidate gene study on pediatric population from North American and Greek-European have suggested that CRP and IL-6 gene polymorphisms was associated with an increased risk OSA in European American but not Greek children. These findings suggest that the IL-6/CRP pathway is associated with OSA [41]. Other candidate gene study has shown an association between SNP within CRP gene and OSA in European Americans and African Americans population [17].

Another study has reported that the polymorphism of *LEPR* gene has a significant correlation with OSA [42]. On the other hand a Japanese study by Hanaoka *et al* [43] has reported that the LEPR gene was not associated with OSAS. It has been suggested that OSA and NAFLD is a multi-factorial and complex disease with a strong genetic basis. The identification of genes implicated through human and animal models in OSA and NAFLD would help to elucidate the pathogenetic processes, which is still largely obscured. In this study we have shown that leptin gene was associated with OSA and NAFLD. Different conclusions in various subject populations may be due to the difference in ethnicity of samples and needs more research. Further studies are required to confirm the association of this gene with the severity of OSA and NAFLD.

The genetics of OSA and NAFLD by genome wide association studies, offers a means of better understanding its pathogenesis with the goals of improving preventive strategies, diagnostic tools and therapies.

One limitation of our study is the lack of data on siblings and other ancestral members of the recruited subjects, which could help in determining the effect of population stratification. We did not have the data on other population controls as well. Clinically it is desirable to identify a marker linked with increased risk of OSA and NAFLD. Also, the diagnosis of NAFLD would be strengthened if, in addition to liver ultrasound, liver biopsy was included, which we could not due to ethical issues. Although, we tried to take care of the sample size within the limited time period of the study, a larger sample size would have further substantiated findings.

## Conclusion

This is the first genetic association of pro-inflammatory cytokine gene (CRP, IL-6 and LEPR) variants in subjects with OSA and NAFLD in Asian Indians residing in north India. These gene polymorphisms were predisposed to the development of OSA and NAFLD. However, these observations need to be evaluated in a larger population for robust results.

## References

[1] Heinzer R, Vat S, Marques-Vidal P, Marti-Soler H, Andries D, Tobback N, et al Prevalence of sleep-disordered breathing in the general population: the HypnoLaus study. Lancet Respir Med. 2015 4;3(4):310–8. 10.1016/S2213-2600(15)00043-0 25682233 PMC4404207

[2] Gami AS, Caples SM, Somers VK. Obesity and obstructive sleep apnea. Endocrinol Metab Clin North Am. 2003 12;32(4):869–94. 14711066 10.1016/s0889-8529(03)00069-0

[3] Musso G, Olivetti C, Cassader M, Gambino R. Obstructive sleep apnea-hypopnea syndrome and nonalcoholic fatty liver disease: emerging evidence and mechanisms. Semin Liver Dis. 2012: 32:49–64. 10.1055/s-0032-1306426 22418888

[4] Minoguchi K, Yokoe T, Tanaka A, Ohta S, Hirano T, Yoshino G et al Association between lipid peroxidation and inflammation in obstructive sleep apnoea. Eur Respir J. 2006; 28:378–385. 10.1183/09031936.06.00084905 16880368

[5] Boston BA. The hypothalamic path to obesity. J Pediatr Endocrinol Metab. 2004;17 Suppl 4:1289–95. 15506075

[6] Savransky V, Nanayakkara A, Vivero A, Li J, Bevans S, Smith PL, et al Chronic intermittent hypoxia predisposes to liver injury. Hepatology. 2007; 45:1007–1013. 10.1002/hep.21593 17393512

[7] Prabhakar N. R., and Kumar. Mechanism of sympathetic activation and blood pressure elevation by intermittent hypoxia. Respir. Physiol. Neurobiol. 2010 11 30;174(1–2):156–61. 10.1016/j.resp.2010.08.021 20804865 PMC3042768

[8] Jun JC, Drager LF, Najjar SS, Gottlieb SS, Brown CD, Smith PL, et al Effects of sleep apnea on nocturnal free fatty acids in subjects with heart failure. Sleep. 2011:1;34(9):1207–13. 10.5665/SLEEP.1240 21886358 PMC3157662

[9] Tilg H, Moschen AR. Evolution of inflammation in nonalcoholic fatty liver disease: the multiple parallel hits hypothesis. Hepatology. 2010;52(5):1836–46. 10.1002/hep.24001 21038418

[10] Majmundar AJ, Wong WJ, Simon MC. Hypoxia-inducible factors and the response to hypoxic stress. Mol Cell. 2010: 22;40(2):294–309. 10.1016/j.molcel.2010.09.022 20965423 PMC3143508

[11] Li J, Bosch-Marce M, Nanayakkara A, Savransky V, Fried SK, Semenza GL Altered metabolic responses to intermittent hypoxia in mice with partial deficiency of hypoxia-inducible factor-1alpha. Physiol Genomics. 2006 5 16;25(3):450–7. Epub 2006 Feb 28. 10.1152/physiolgenomics.00293.2005 16507783

[12] Foretz M, Pacot C, Dugail I, Lemarchand P, Guichard C, Le Lièpvre X ADD1/SREBP-1c is required in the activation of hepatic lipogenic gene expression by glucose. Mol Cell Biol. 1999;19(5):3760–8. 10207099 10.1128/mcb.19.5.3760 PMC84202

[13] Biddinger SB, Almind K, Miyazaki M, Kokkotou E, Ntambi JM, Kahn CR. Effects of diet and genetic background on sterol regulatory element-binding protein-1c, stearoyl-CoA desaturase 1, and the development of the metabolic syndrome. Diabetes. 2005;54(5):1314–23. 15855315 10.2337/diabetes.54.5.1314

[14] Bhushan B, Guleria R, Misra A, Pandey RM, Luthra K, Vikram NK. Obstructive sleep apnoea correlates with C-reactive protein in obese Asian Indians. Nutr Metab Cardiovasc Dis. 2009; 19:184–9. 10.1016/j.numecd.2008.06.008. Epub 2008 Sep 20. 18805681

[15] Yudkin JS, Kumari M, Humphries SE, Mohamed-Ali V.Inflammation, obesity, stress and coronary heart disease: is IL-6 the link? Atherosclerosis. 2000 2 148:209–14. 10657556 10.1016/s0021-9150(99)00463-3

[16] Heinrich PC, Castell JV, Andus T. Interleukin-6 and the acute phase response. Biochem J. 1990 2 1;265(3):621–36. 1689567 10.1042/bj2650621 PMC1133681

[17] Larkin EK, Patel SR, Goodloe RJ, Li Y, Zhu X, Gray-McGuire C, et al A candidate gene study of obstructive sleep apnea in European Americans and African Americans. Am J Respir Crit Care Med. 2010 10 1;182(7):947–53. 10.1164/rccm.201002-0192OC. Epub 2010 Jun 10. 20538960 PMC2970865

[18] Misra A and Vikram N. K., “Insulin resistance syndrome (metabolic syndrome) and Asian Indians,” Current Science, vol. 83, no. 12, pp. 1483–1496, 2002.

[19] Dudeja V, Misra A, Pandey RM, Devina G, Kumar G, Vikram NK.BMI does not accurately predict overweight in Asian Indians in Northern India. Br J Nutr 2001 7;86(1):105–12. 11432771 10.1079/bjn2001382

[20] Bhatt SP, Nigam P, Misra A, Guleria R, Qadar Pasha MA.Independent associations of low 25 hydroxy vitamin D and high PTH levels with non alcoholic fatty liver disease in Asian Indians residing in north India. Athroscorosis; 2013 Sep;230(1):157–63. 10.1016/j.atherosclerosis.2013.07.006. Epub 2013 7 22. 10.1016/j.atherosclerosis.2013.07.006 23958268

[21] Bajaj P. Nigam, A. Luthra, R.M. Pandey, D. Kondal, S.P. Bhatt, et al A case-control study on insulin resistance, metabolic co-variates & prediction score in non-alcoholic fatty liver disease. Indian J Med Res. 2009 3;129(3):285–92. 19491421

[22] Tam KM, Wu JS. Ultrasonographic diagnosis of fatty liver. Taiwan Yi Xue Hui Za Zhi 1986; 85:45–53. 3519848

[23] BaHammam AS, Obeidat A, Barataman K, Bahammam SA, Olaish AH, Sharif MM. A comparison between the AASM 2012 and 2007 definitions for detecting hypopnea. Sleep Breath. 2014 12;18(4):767–73. 10.1007/s11325-014-0939-3. Epub 2014 Feb 4. 24493077

[24] Martínez-Calleja, Quiróz-Vargas Irma, Parra-Rojas Isela, Muñoz-Valle José Francisco et al Haplotypes in the CRP Gene Associated with Increased BMI and Levels of CRP in Subjects with Type 2 Diabetes or Obesity from Southwestern Mexico American. Experimental Diabetes Research. 2012;2012:982683 10.1155/2012/982683. Epub 2012 Sep 25. 23049543 PMC3463182

[25] Murugesan Devi, Arunachalam Thirunavukkarasu, Ramamurthy Viraragavan, and Subramanian Selvi. Association of polymorphisms in leptin receptor gene with obesity and type 2 diabetes in the local population of Coimbatore.Indian J Hum Genet. 2010 5;16(2):72–7. 10.4103/0971-6866.69350 21031055 PMC2955955

[26] Gotoda T, Manning BS, Goldstone AP. Leptin receptor gene variation and obesity: Lack of association in a white British male population. Hum Mol Genet. 1997 6; 6:869–76 9175732 10.1093/hmg/6.6.869

[27] Venkat Reddy Tumu Suresh Govatati, Guruvaiah Praveen, Deenadayal Mamata, Shivaji Sisinthy, Bhanoori Manjula. An interleukin-6 gene promoter polymorphism is associated with polycystic ovary syndrome in South Indian women. J Assist Reprod Genet. 2013 12;30(12):1541–6. 10.1007/s10815-013-0111-1. Epub 2013 Oct 10. 24114630 PMC3843174

[28] Misra A, Chowbey P, Makkar BM, Vikram NK, Wasir JS, Chadha D, et al Consensus statement for diagnosis of obesity, abdominal obesity and the metabolic syndrome for Asian Indians and recommendations for physical activity, medical and surgical management. J Assoc Physicians India 2009; 57: 163–70. 19582986

[29] Expert Committee on the diagnosis and classification of Diabetes Mellitus; (2003) Report of the expert committee on the diagnosis and classification of diabetes mellitus. Diabetes Care003 1;26 Suppl 1:S5–20. 12502614 10.2337/diacare.26.2007.s5

[30] Lambert M, Paradis G, O'Loughlin J, Delvin EE, Hanley JA, Levy E. Insulin resistance syndrome in a representative sample of children and adolescents from Qubec, Canada. Int J Obes Relat Metab Disord 2004 7; 28: 833–841. 10.1038/sj.ijo.0802694 15170466

[31] Homeostasis model assessment: insulin resistance and beta-cell function from fasting plasma glucose and insulin concentrations in man. Diabetologia 1985 7; 28: 412–419. 3899825 10.1007/BF00280883

[32] Arnold Joseph, Sunilkumar M., Krishna V., Yoganand S. P., Sathish Kumar M., and Shanmugapriyan D. Obstructive Sleep Apnea. J Pharm Bioallied Sci. 2017 11;9(Suppl 1):S26–S28. 10.4103/jpbs.JPBS_155_17 29284930 PMC5731026

[33] Heinrich PC, Castell JV, Andus. T Interleukin-6 and the acute phase response. Biochem J. 1990 2 1; 265(3):621–36. 1689567 10.1042/bj2650621 PMC1133681

[34] Raji A, Seely EW, Arky RA, Simonson DC. Body fat distribution and insulin resistance in healthy Asian Indians and Caucasians. J Clin Endocrinol Metab. 2001 11; 86: 5366–5371. 10.1210/jcem.86.11.7992 11701707

[35] Misra A, Khurana L, Obesity and the metabolic syndrome in developing countries. J Clin Endocrinol Metab. 2008 11;93(11 Suppl 1):S9–30. 10.1210/jc.2008-1595 18987276

[36] Rajendran K, Devarajan N, Ganesan M, Ragunathan M. Obesity, Inflammation and Acute Myocardial Infarction—Expression of leptin, IL 6 and highsensitivity-CRP in Chennai based population. Thromb J. 2012: 14 8; 10(1):13. 10.1186/1477-9560-10-13 PMC3444897 22891684

[37] Ryan S, Taylor CT, McNicholas WT: Selective activation of inflammatory pathways by intermittent hypoxia in obstructive sleep apnea syndrome.Circulation, 2005; 112: 2660–2667. 10.1161/CIRCULATIONAHA.105.556746 16246965

[38] Santos-Alvarez J, Goberna R, Sánchez-Margalet V Human leptin stimulates proliferation and activation of human circulating monocytes. Cell Immunol. 1999 5 25; 194(1):6–11. 10.1006/cimm.1999.1490 10357875

[39] Haddy N, Sass C, Droesch S, Zaiou M, Siest G, Ponthieux A, Lambert D, Visvikis S L-6, TNF-alpha and atherosclerosis risk indicators in a healthy family population: the STANISLAS cohort. Atherosclerosis. 2003 10; 170(2):277–83. 14612208 10.1016/s0021-9150(03)00287-9

[40] Karaduman M, Oktenli C, Musabak U, Sengul A, Yesilova Z, Cingoz F et al Leptin, soluble interleukin-6 receptor, CRP and soluble vascular cell adhesion molecule 1 levels in human coronary atherosclerotic plaque. Clin Exp Immunol. 2006 3; 143(3):452–7. 10.1111/j.1365-2249.2006.03025.x 16487244 PMC1809610

[41] Karaduman M, Oktenli C, Musabak U, Sengul A, Yesilova Z, Cingoz F. Variants in C reactive protein and IL-6 genes and susceptibility to obstructive sleep apnea in children: a candidate-gene association study in European American and Southeast European populations. Sleep Med. 2014: 15:228–235. 10.1016/j.sleep.2013.08.795 24380782 PMC3940266

[42] Popko K, Gorska E, Wasik M, Plywaczewski R, Winiarska M, Gorecka D, et al Frequency of distribution of leptin receptor gene polymorphism in obstructive sleep apnea patients. J Physiol Pharmacol 2007; 58 Suppl 5: 551–561. 18204169

[43] Hanaoka M, Yu X, Urushihata K, Ota M, Fujimoto K, Kubo K. Leptin and leptin receptor gene polymorphisms in obstructive sleep apnea syndrome. Chest. 2008; 133(1):79–85. Epub 2007 Nov 7. 10.1378/chest.07-1633 17989154

