# Correction: Association of inflammatory genes in obstructive sleep apnea and non alcoholic fatty liver disease in Asian Indians residing in north India

**DOI:** 10.1371/journal.pone.0203182

**Published:** 2018-08-23

**Authors:** Surya Prakash Bhatt, Randeep Guleria, Naval K. Vikram, S. Vivekanandhan, Yogendra Singh, A. K. Gupta

The fourth author’s name is spelled incorrectly. The correct name is: S. Vivekanandhan. The correct citation is: Bhatt SP, Guleria R, Vikram NK, Vivekanandhan S, Singh Y, Gupta AK (2018) Association of inflammatory genes in obstructive sleep apnea and non alcoholic fatty liver disease in Asian Indians residing in north India. PLoS ONE 13(7): e0199599. https://doi.org/10.1371/journal.pone.0199599
